# Supplementary material for: A Succession of Microbiome Communities in the Early Establishing Process of an Epilithic Algal Matrix in a Fringing Reef
Source: Microorganisms. 2025 Mar 17;13(3):672. doi: 10.3390/microorganisms13030672 (PMC11944468; doi:10.3390/microorganisms13030672)
Supplement: Supplementary file 1 [file microorganisms-13-00672-s001.zip › microorganisms-3450936-supplementary.pdf]

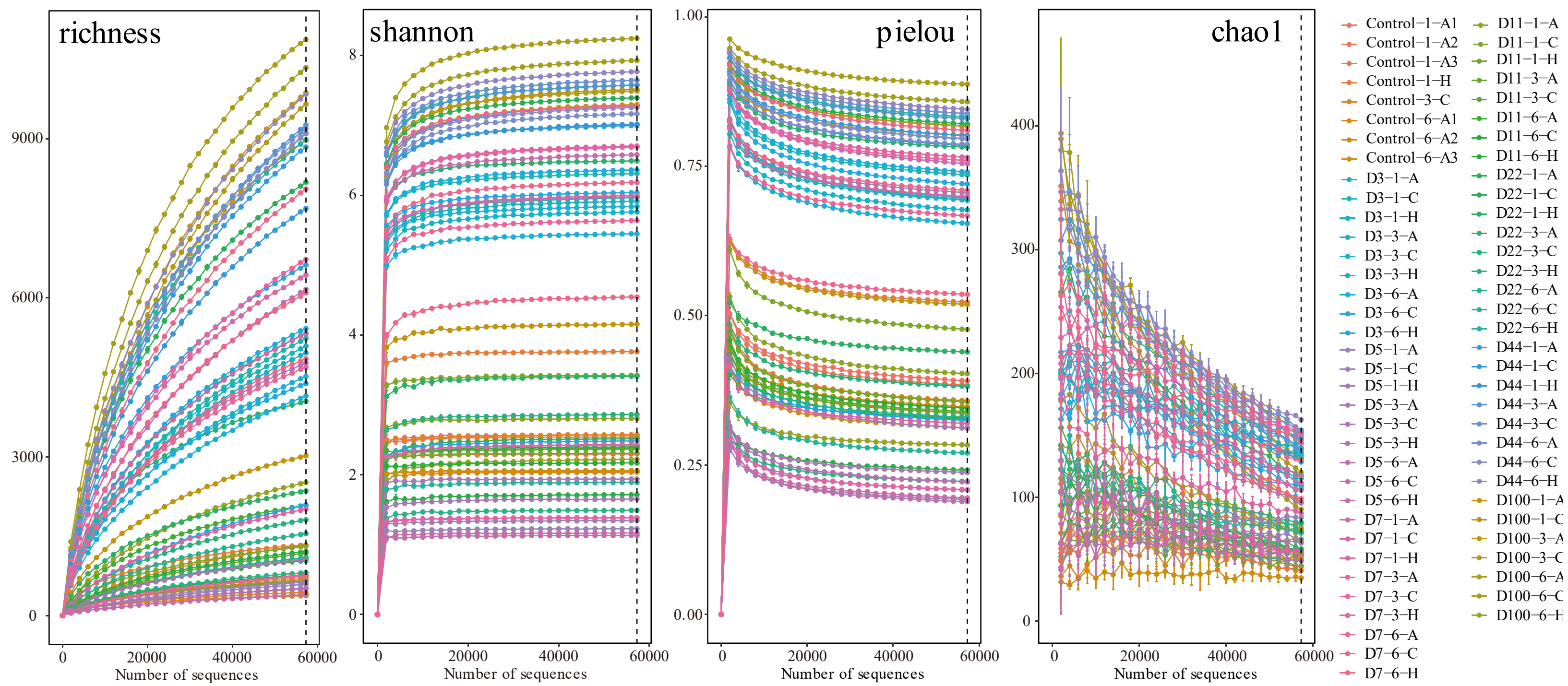

Figure S1: Rarefaction curve

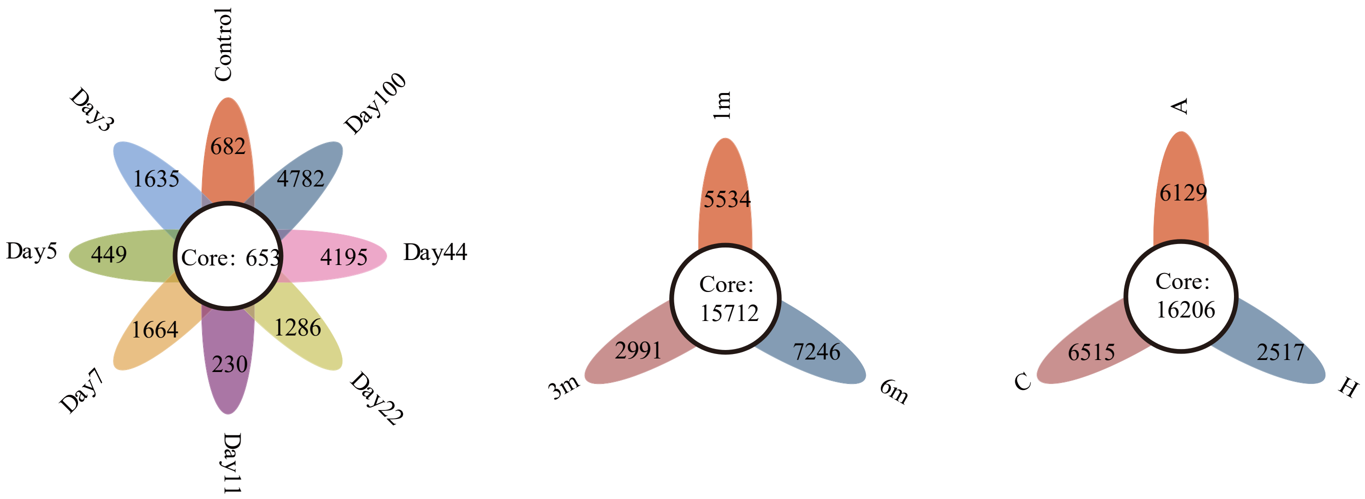

Figure S2: Venn diagram

Table S1 Sample collection results

Group		Day3	Day5	Day7	Day11	Day22	Day44	Day100	Control
Deep-1m	Type-A	1	1	1	1	1	1	1	3
	Type-C	1	1	1	1	1	1	1	——
	Type-H	1	1	1	1	1	1	——	1
Deep-3m	Type-A	1	1	1	1	1	1	1	——
	Type-C	1	1	1	1	1	1	1	1
	Type-H	1	1	1	——	1	——	——	——
Deep-6m	Type-A	1	1	1	1	1	1	1	3
	Type-C	1	1	1	1	1	1	1	——
	Type-H	1	1	1	1	1	1	1	——

Table S2 Functional taxa

functional taxa	taxonomy	reference
probioces	*Endozoicomonas*	(Neave et al., 2017)
	Pseudoalteromonas	(Maya Shnit-Orland, Alex Sivan, & Ariel Kushmaro, 2012)
	Ralstonia	(Katie L. Barott & Forest L. Rohwer, 2012)
potential pathogens	*Photobacterium*	(Austin, 2010)
	Alteromonas	(Rosenberg, Koren, Reshef, Efrony, & Zilber-Rosenberg, 2007)
	Pseudomonas	(Stover et al., 2000)
	Thalassotalea	(Rosenberg, Koren, Reshef, Efrony, & Zilber-Rosenberg, 2007)
	Kiloniellaceae	(Rosenberg, Koren, Reshef, Efrony, & Zilber-Rosenberg, 2007)
	Bacillus	(Vilas-Boas et al., 2007)
pathogenics	*Ruegeria*	(Liew et al., 2018)
	Serratia	(Rosenberg, Koren, Reshef, Efrony, & Zilber-Rosenberg, 2007)
	Vibrio	(Rosenberg, Koren, Reshef, Efrony, & Zilber-Rosenberg, 2007)
	Beggiatoales	(Rosenberg, Koren, Reshef, Efrony, & Zilber-Rosenberg, 2007)
	Desulfovibrio	(Liu and Haggblom, 2018)
	Phormidium	(Rosenberg, Koren, Reshef, Efrony, & Zilber-Rosenberg, 2007)
autotrophic	Cyanobacteria	（El-Seedi et al., 2023)
	Nunduva	(Gonzalez-Resendiz et al., 2018)
	Neolyngbya	(Nuryadi and Suda, 2022)
	Chroococcidiopsis	(Cumbers and Rothschild, 2014)

Table S3 Multifactor analysis of variance (ANOVA)

source	implicit variable	Type III sum of squares	df	R^2	F	Sig
modified model	Richness	57671.241[a]	61	945.43	5.184	0.036
	Shannon	73.642[b]	61	1.207	4.541	0.047
	Pielou	2.698[d]	61	0.044	4.914	0.04
	Chao1	56942.116[f]	61	933.477	3.042	0.106
intercept	Richness	456101.33	1	456101.33	2501.013	0
	Shannon	348.035	1	348.035	1309.166	0
	Pielou	16.854	1	16.854	1872.274	0
	Chao1	568519.739	1	568519.739	1852.602	0
Day	Richness	29964.918	7	4280.703	23.473	0.002
	Shannon	29.552	7	4.222	15.88	0.004
	Pielou	0.995	7	0.142	15.789	0.004
	Chao1	26325.591	7	3760.799	12.255	0.007
depth	Richness	32.495	2	16.247	0.089	0.916
	Shannon	0.09	2	0.045	0.169	0.849
	Pielou	0.003	2	0.002	0.177	0.843
	Chao1	232.5	2	116.25	0.379	0.703
type	Richness	1330.234	2	665.117	3.647	0.105
	Shannon	7.585	2	3.792	14.265	0.009
	Pielou	0.319	2	0.16	17.734	0.005
	Chao1	1294.644	2	647.322	2.109	0.217
Day * depth	Richness	2460.256	13	189.25	1.038	0.525
	Shannon	3.341	13	0.257	0.967	0.562
	Pielou	0.135	13	0.01	1.158	0.469
	Chao1	3744.573	13	288.044	0.939	0.577
Day * type	Richness	6151.951	13	473.227	2.595	0.15
	Shannon	9.733	13	0.749	2.816	0.13
	Pielou	0.374	13	0.029	3.194	0.104
	Chao1	6450.472	13	496.19	1.617	0.312
depth * type	Richness	342.036	4	85.509	0.469	0.759
	Shannon	0.09	4	0.023	0.085	0.983

	Pielou	0.004	4	0.001	0.101	0.977
	Chao1	698.323	4	174.581	0.569	0.698
	Richness	9808.079	19	516.215	2.831	0.126
	Shannon	19.576	19	1.03	3.876	0.07
	Pielou	0.756	19	0.04	4.422	0.053
	Chao1	9447.315	19	497.227	1.62	0.312
difference	Richness	911.833	5	182.367		
	Shannon	1.329	5	0.266		
	Pielou	0.045	5	0.009		
	Chao1	1534.382	5	306.876		
total	Richness	591785	67			
	Shannon	487.699	67			
	Pielou	22.799	67			
	Chao1	719270.089	67			
revised total	Richness	58583.075	66			
	Shannon	74.971	66			
	Pielou	2.743	66			
	Chao1	58476.497	66			

a. $R2 = 0.984$ (revised $R2 = 0.795$)

b. $R2 = 0.982$ (revised $R2 = 0.766$)

c. $R2 = 0.993$ (revised $R2 = 0.902$)

d. $R2 = 0.984$ (revised $R2 = 0.783$)

e. $R2 =0.956$ (revised $R2 = 0.423$)

f. $R2 = 0.974$ (revised $R2 = 0.654$)

g. $R2 = 0.981$ (revised $R2 = 0.745$)

h. $R2 = 0.993$ (revised $R2 = 0.903$)

Table S4 ADONIS test on deep and type in different succession stages

Parameters	Stage	Variable	R^2	*p* value	Sig
OTUs	Early	deep	0.041	0.483	
		type	0.056	0.239	
	Median	deep	0.193	0.043	*
		type	0.121	0.782	
Functional taxa	Early	deep	0.027	0.614	
		type	0.076	0.163	
	Median	deep	0.139	0.451	
		type	0.209	0.088	
Predicted function	Early	deep	0.03	0.716	
		type	0.025	0.802	
	Median	deep	0.098	0.671	
		type	0.234	0.113	

Table S5 Attributes of the co-occurrence network

Parameter	Early stage	Median stage	Later stage
Nodes	197	186	110
Edges	4453	1084	722
Average degree	45.208	11.656	13.127
Average weighted degree	26.97	9.646	12.747
Network diamete	6	12	6
Density	0.231	0.063	0.12
Modularity	0.355	0.682	0.487
Weakly connected components	3	3	12
Average clustering coefficient	0.738	0.664	0.894
Average path length	2.199	4.359	2.122

Table S6 Summary of stepwise regression analysis parameter estimation results

level	Dependent variable	Variable	Parameter estimate	Standard error	*t* value	*P*r > F
alpha-diversity	Richness	deep	0.495	565.407	3.097	0.006**
		temperature	0.8	1593.997	5.01	0.001**
	Shannon	deep	0.394	0.434	2.165	0.044*
		temperature	0.729	1.224	4.005	0.001**
	Pielou	temperature	0.555	0.127	2.912	0.009**
	Chao1	deep	0.494	764.216	3.074	0.007**
		temperature	0.797	2154.478	4.957	0.000**
phylum	Bacteroidota	deep	0.441	1232.457	2.513	0.022*
		temperature	0.75	5909.983	4.275	0.001**
	Bdellovibrionota	deep	0.593	45.225	3.761	0.001**
		temperature	0.765	127.498	4.849	0.001**
	Verrucomicrobiota	light intensity	-0.4	0.042	-2.319	0.032*
		temperature	0.802	253.261	4.649	0.001**
	Cyanobacteria	light intensity	0.546	0.646	2.838	0.011*
genus	*Photobacterium*	temperature	-0.556	4532.774	-2.918	0.009**
	Pseudoalteromonas	temperature	0.537	470.864	2.776	0.012*
functional taxa	Probioces	temperature	0.002	0.002	3.106	0.006**
	Autotrophics	light intensity	0.546	0.646	2.838	0.011*
	Potential pathogenics	temperature	-0.0632	0.058	-3.475	0.003*
predicted function	aerobic chemoheterotrophy	temperature	-0.503	10071.58	-2.535	0.020*
	chloroplasts	light intensity	0.534	0.648	2.754	0.013*
	chemoheterotrophy	temperature	-0.501	10081.485	-2.526	0.021*
